# Greenspace redevelopment, pressure of displacement, and sleep quality among Black adults in Southwest Atlanta

**DOI:** 10.1038/s41370-021-00313-9

**Published:** 2021-03-13

**Authors:** Patrice C. Williams, Robert Krafty, Terrence Alexander, Zipporah Davis, Akil-Vuai Gregory, Raven Proby, Wendy Troxel, Christopher Coutts

**Affiliations:** 1 Department of Urban & Regional Planning, Florida State University, Tallahassee, FL USA; 2 Department of Biostatistics and Bioinformatics, Rollins School of Public Health, Emory University, Atlanta, GA USA; 3 Master of Public Health Program, Morehouse School of Medicine, Atlanta, GA USA; 4 RAND Corporation, Pittsburgh, PA USA

**Keywords:** Greenspace redevelopment, Sleep quality, Social environmental stressors, Atlanta BeltLine, Black adults, Pressure of displacement

## Abstract

**Background:**

Little is known on how greenspace redevelopment—creating or improving existing parks and trails—targeted for low-income and/or majority Black neighborhoods could amplify existing social environmental stressors, increase residents’ susceptibility to displacement, and impact their sleep quality.

**Objective:**

To examine the relationship between social environmental stressors associated with displacement and sleep quality among Black adults.

**Methods:**

Linear regression models were employed on survey data to investigate the association between social environmental stressors, independently and combined, on sleep quality among Black adults residing in block groups targeted for greenspace redevelopment (i.e., exposed) and matched with block groups that were not (i.e., unexposed).

**Results:**

The independent associations between everyday discrimination, heightened vigilance, housing unaffordability, and subjective sleep quality were not modified by greenspace redevelopment, controlling for other factors. The association between financial strain and subjective sleep quality was different for exposed and unexposed participants with exposed participants having a poorer sleep quality. The combined model revealed that the association between financial strain and sleep quality persisted. However, for different financial strain categories exposed participants slept poorer and/or better than unexposed participants.

**Significance:**

Our findings suggest a nuanced relationship between social environmental stressors, pressure of displacement related to greenspace redevelopment, and sleep quality among Black adults.

## Introduction

Parks and greenspaces are associated with physical and mental health benefits, such as more active lifestyles [1, 2], lower stress levels [3], higher sleep duration [4], improved functioning of our natural ecosystem and decreases in air pollution [5], as well as a means to maintain neighborhood social ties [1]. In general, individuals in low-income neighborhoods and neighborhoods that predominantly consist of Black, Indigenous, and People of Color (BIPOC) have lived with inequitable environmental quality, where they are more likely to have limited access to environmental amenities (e.g., parks, trails, community gardens) [6–9] and increased exposure to environmental pollutants [10, 11]. Residential segregation has been a key mechanism by which racial inequality has been created and reinforced because it shapes the socioeconomic conditions for BIPOC. Therefore, greenspace redevelopment—creating or improving existing parks, trails, and greenspaces—is an environmental justice issue because it is generally not equitably distributed. As municipalities move toward strategies that are focused on greenspace redevelopment, it is important to examine the impact of these decisions from an equity lens.

Green initiatives that promote either increasing the number of parks, green roofs, as well as building infrastructure for alternative modes of transportation have the unintentional consequence of inciting and/or enabling the process of “green gentrification”—a process that creates or restores environmental amenities, increases property values, and displaces socially vulnerable populations [12]. Although residential displacement is a central concern of gentrification, it is not a phenomenon that is distinct to gentrification. Grier and Grier [13] define residential displacement as conditions that affect the home or its immediate surroundings that “1) are beyond the household’s reasonable ability to control or prevent; 2) occur despite the household’s having met all previously imposed conditions of occupancy; and 3) make continued occupancy by that household impossible, hazardous, or unaffordable” (p. 8). Marcuse [14] conceptualizes three of the four forms of displacement as economic displacement (e.g., due to increases in rent), exclusionary displacement (e.g., related to unaffordable housing and loss of social networks), and displacement pressure, which he characterizes as the combination of subjective fear of being displaced and what is actually occurring in the neighborhood (e.g., increases in rent and property taxes and loss of cultural significance of a place). Thus, the municipal intervention of greenspace redevelopment will impact the existing neighborhood conditions and increase the susceptibility of low-income and BIPOC residents to residential displacement.

**Fig. 1:**
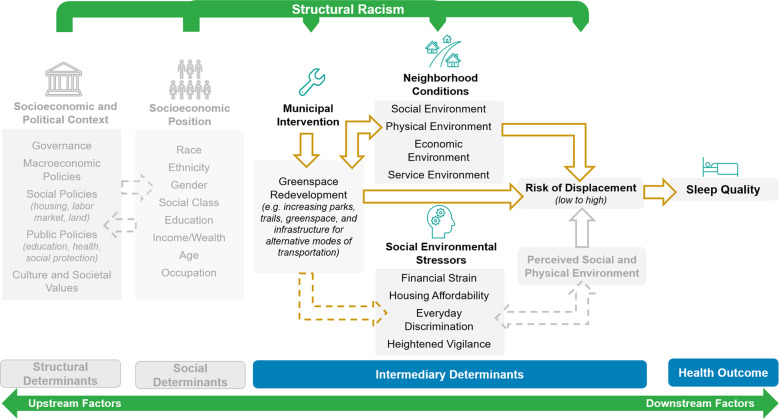
Conceptual framework of sleep quality among Black residents as an outcome of greenspace redevelopment. Grayed text and arrows: dynamic structural and social factors that are influencing individual- and population-level risk of displacement and sleep quality. Broken arrows: interrelations taking place. Solid arrows: potential pathways of how greenspace redevelopment and social and environmental stressors can impact sleep quality.

Black individuals are disproportionately likely to live in disinvested neighborhoods because of residential segregation, which creates social and physical risks in their residential environments that adversely affect health [15]. Researchers have argued that BIPOC experience greater levels of social stressors and report higher levels of psychosocial stress [16]. When Black neighborhoods undergo greenspace redevelopment, it could amplify existing social environmental stressors, such as discrimination, heightened vigilance, housing unaffordability, financial strain, which are also associated with displacement (Fig. 1). Discrimination in the context of greenspace redevelopment can be experienced in terms of social exclusion from the newly created spaces and erasure of their community likeness [17–20]. In response, it can stimulate a heightened awareness and vigilance to protect oneself from anticipated discrimination [21]. Housing unaffordability and financial strain can be experienced by residents who do not have the financial capacity to maintain current expenses and absorb substantial increases in rent and property taxes due to rapid housing appreciation rates [19, 20, 22, 23]. Research suggests that chronic stress resulting from rumination and self-preservation is associated with poor sleep outcomes [24, 25].

Sleep disturbances have been associated with chronic stressors, such as financial stress [26, 27] and housing instability [28]. Moreover, studies have shown a positive association between discrimination or unfair treatment and sleep difficulties and shorter sleep duration [29, 30]. Feagin’s [31] ethnographic study documented that Black individuals reported not only prior experiences with discrimination but also prepared for the possibility of future experiences with discrimination. Researchers have termed these thoughts and behaviors as “racism-related vigilance” [32]. Hicken et al. [33] found that Black individuals reported greater levels of vigilance and sleep difficulty compared to White individuals.

This study used the Atlanta BeltLine, as the greenspace redevelopment project of interest, to examine the relationship between social environmental stressors associated with residential displacement and sleep quality among Black adults. The Atlanta BeltLine is a public–private greenspace redevelopment project that initiated in 2005 and will result in improvements to 700 acres of existing parks, the addition of 1300 acres of new and expanded greenspace, 33 miles of new multiuse trails, and a 22-mile loop of rail transit service by 2030. Two census block groups, within the Atlanta BeltLine target development area, classified as medium to high-risk areas of residential displacement were matched using propensity score matching to block groups within the City of Atlanta that were unexposed to the Atlanta BeltLine to examine the relationship between social environmental stressors and subjective sleep quality among Black adults. We hypothesized the following: (1) social environmental stressors, in individual and combined models, will be correlated with poorer subjective sleep quality; (2) the association between social environmental stressors and subjective sleep quality will differ for participants exposed to greenspace redevelopment (i.e., areas classified as medium to high-risk of displacement) compared to participants unexposed to greenspace redevelopment; and (3) participants exposed to greenspace redevelopment will have poorer subjective sleep quality.

## Methods

### Study design and procedures

The African American Sleep & Health (AASH) Study was a pilot, cross-sectional, quasi-experimental study where the primary objective was to understand the relationships between neighborhoods exposed and unexposed to greenspace redevelopment, social environmental stressors associated with residential displacement, and sleep quality among African American/Black adults. First, we used a structural racism lens to develop a displacement risk index, discussed elsewhere [34], to identify census block groups within the target development area of the Atlanta BeltLine that have a concentration of residents who are most susceptible to the pressure of displacement. Briefly, each block group was assigned a composite displacement risk score calculated using *z*-scores to compare its performance in each of the vulnerability and housing market indicators (Supplementary Table 1) relative to the City of Atlanta’s estimate over time (i.e., 2000, 2007–2011, 2012–2016). For each timepoint the composite score for the vulnerability and housing market indicators were added together, linearly transformed (i.e., to create only positive scores within the index), and reclassified using quartiles, which ranged the displacement susceptibility levels from low to high risk of displacement. Second, we used propensity score matching to pair exposed (i.e., block groups characterized as medium to high-risk of displacement) to unexposed block groups. Third, we employed a survey to eligible households within the exposed and unexposed block groups that asked questions pertaining to social environmental stressors (i.e., everyday discrimination, heightened vigilance, housing unaffordability, and financial strain), subjective sleep quality, and sociodemographic covariates. Institutional review board approval was granted at Florida State University and Morehouse School of Medicine with Florida State University being the designated institutional review board. Written informed consent was obtained from all participants.

### Identifying exposed and unexposed block groups to greenspace redevelopment

Two census block groups, within the target development area of the Atlanta BeltLine, characterized as medium to high-risk of residential displacement were selected as exposed block groups for the following reasons: (1) we observed that over time the median rents, percent of severely cost-burdened households, and eviction, crime, and vacancy rates were higher than the City of Atlanta, which previous studies have shown are correlated with disinvestment- and investment-related displacement [14, 35–38], (2) The American Community Survey 2012–2016 estimates showed that these block groups had a high percent (100%) of non-Hispanic African American/Black residents (Supplementary Tables 2 and 3) the 3-mile multiuse Westside trail of the Atlanta BeltLine, which initiated in fall 2014 and completed in September 2017, is located in these block groups.

**Fig. 2:**
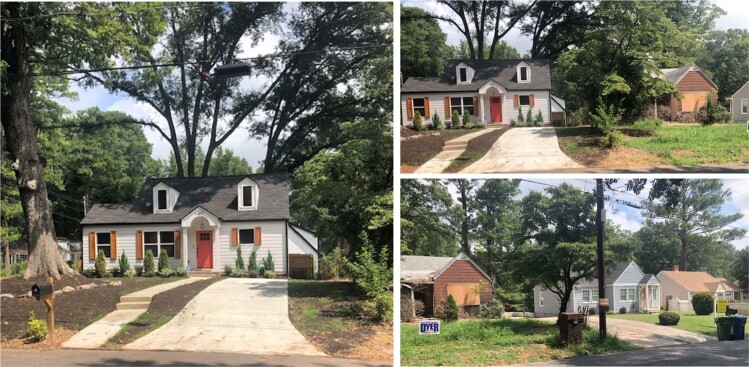
Example of properties in the exposed block groups. Left panel: rehabilitated property. Top right panel: rehabilitated property adjacent to an abandoned property. Bottom right panel: abandoned property next to rehabilitated property that is for sale. Photo credit: co-author TA.

Prior studies have demonstrated that soon after publicly invested projects secure funding or the planning process is announced in local media, housing prices increases substantially in the surrounding neighborhoods [23, 39]. This increase could be attributed to in-movers willing to pay a premium for housing near the new project and placing current residents at risk of displacement. Immergluck and Balan [40] found that neighborhoods along the southwest segment of the BeltLine (i.e., the location of our two exposed block groups) saw the highest median sale prices increase—at 68% compared to 40% within the southeast and northeast segments, 51% within the northwest segment, or 17.7% across the rest of the city from 2011 to 2015. Therefore as seen in other redevelopment projects [12, 41], we anticipated a decrease in Black residents and an increase in White residents within our exposed block groups when we initiated the study in May 2019. In addition, when the research team visited both block groups we noted the physical signs of disinvestment and reinvestment, meaning there were adjacent properties that were either abandoned (e.g., boarded windows), undergoing rehabilitation (e.g., construction crew working on the property), newly constructed and for sale or recently purchased, or an older occupied property (example in Fig. 2). Although social environmental stressors already exist in these communities due to factors such as structural racism and residential segregation, we believe that neighborhood changes related to greenspace redevelopment within our exposed block groups could amplify these social environmental stressors—discrimination, heightened vigilance, housing unaffordability, and financial strain—for current residents, which could increase their susceptibility to the pressure of displacement compared to residents of block groups that are not exposed to greenspace redevelopment.

**Fig. 3:**
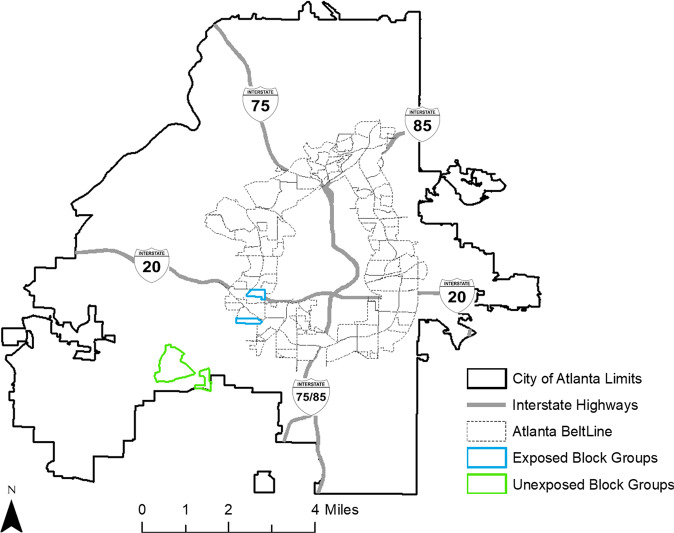
Study recruitment areas. Map of exposed and unexposed block groups selected for AASH (June–October 2019).

Propensity score matching forms matched sets of exposed (i.e., block groups characterized as medium to high-risk of displacement) and unexposed block groups that have balanced on a large number of covariates [42]. We selected indicators from the 2012–2016 American Community Survey 5-year estimates to measure the neighborhood economic and racial compositions and population and housing characteristics (Supplementary Table 2). Prior research has shown that these indicators provide insight on the characteristics of neighborhoods experiencing changes due to redevelopment [22, 43]. Propensity scores were estimated for each block group using the percent below poverty as the outcome variable because sleep data were not available at the block group level. The matching procedure was conducted using “teffects psmatch” command in Stata. The exposed block groups were matched 3:1 with replacement (to yield the largest number of matched pairs) to unexposed block groups with propensity scores within a range of ±0.01 (Fig. 3). To minimize possible bias from spatial spillovers from greenspace redevelopment, we excluded census block groups located within 1 mile of the exposed block groups from consideration. Propensity score results are displayed in Supplementary Table 3. Box plots show there is overlap in the distribution of propensity scores in the exposed and unexposed block groups (Supplementary Fig. 1). Descriptive characteristics of the exposed and unexposed block groups selected for this study are displayed in Supplementary Table 2.

### Sampling

We utilized a two-stage cluster sampling approach involving the random selection of blocks (primary sampling units) within census block groups (*n* = 4; 2 exposed and 2 unexposed) and households (secondary sampling units) within blocks. The sampling frame for this study was 374 households (143 exposed and 231 exposed). The number of recruited households differed for exposed and unexposed block groups because the unexposed block groups had larger blocks and hardly any abandoned properties. In addition, one of the unexposed block groups was mainly apartment complexes. Therefore, we recruited from an apartment complex that had 100% occupancy and 98% of its tenants were Black.

Maps for the census block groups and for each randomly selected block were prepared and used to randomly select one of the four corners of a block; the household closest to the selected corner was identified as the starting point. Next, using aerial maps, team members proceeded from the starting point in a clockwise direction and recruited every third household (or third building within the apartment complex) in each block to ensure objectivity in household selection. If the house appeared abandoned, for sale, or an Airbnb property—these were identified by a neighbor, current tenant, or had a keyless door lock—it was not included in the sampling and we chose an adjacent house.

### Recruitment

Each randomly selected household was contacted up to five times by a combination of methods to request their voluntary participation. During the first stage of recruitment, team members traveled in pairs to ensure their safety and dropped off an advanced letter and flyer notifying residents of the study. The advanced letter included a link to the study’s website that provided additional information about the study and prescreening questions on Qualtrics (Qualtrics, Provo, UT), which included questions on their self-identified race, age, gender, previous sleep disorder diagnosis, and whether they are head of household or one of the joint financial decision-makers for the household. If eligible, they were asked to select a preferable day and time for data collection upon consent. On the second household visit, team members knocked on the doors of the randomly selected homes. If contact was not made, a second letter was left. Subsequent visits were made as needed. The times and days of the week for these visits were systematically varied to maximize the chance of contact.

When contact was made, interested adults (18 years or older) were verbally consented prior to administering the online prescreening questionnaire, which determined whether they were eligible for the study. Recruited adults were ineligible for the study if (1) they did not self-identify as African American/Black, (2) were previously diagnosed with a sleep condition, and/or (3) were not the head of household or one of the joint financial decision-makers for the household. After screening, eligible participants were consented prior to administering the online survey using SurveyMonkey (SurveyMonkey, San Mateo, CA). All participants received a $25 gift card upon completion. Survey data were collected from June 2019 to October 2019. We use listwise deletion to handle all missing data (<3.03% for all variables except financial strain (31.8%)). The sample size for housing affordability is smaller due to these questions only being asked to renters and not homeowners. The final sample included 66 (42 unexposed and 24 exposed) Black adults, which represented 18% of the residents’ approached for participation. Each participant was assigned a survey number to protect their anonymity.

### Subjective sleep quality

Subjective sleep quality was measured with the Pittsburgh Sleep Quality Index (PSQI) [44], a 19-item questionnaire that measures aspects of sleep over the preceding month. Questions inquire about sleep duration, sleep disturbance, sleep latency (i.e., time spent falling asleep), dysfunction during the day due to sleepiness (i.e., trouble staying awake while driving), sleep efficiency (i.e., time in bed spent asleep), and sleep medication use. Composite scores on the PSQI range from 0 to 21, scores of 5 and greater on the PSQI indicate clinically significant poor sleep quality [44]. The PSQI has been widely used across study populations with good validity and high test–retest reliability [45].

### Social environmental stressors

Everyday discrimination was measured using nine questions [46]. Respondents were asked in their day-to-day lives, how often do any of the following things happen to them: (1) you were treated with less courtesy than other people are, (2) you are treated with less respect than other people are, (3) you receive poorer service than other people at restaurants or stores, (4) people act as if they think you are not smart, (5) people act as if they are afraid of you, (6) people act as if they think you are dishonest, (7) people act as if they are better than you are, (8) you are called names or insulted, and (9) you are threatened or harassed. Responses were on a Likert-like scale of 1 = almost everyday, 2 = at least once a week, 3 = a few times a month, 4 = a few times a year, 5 = less than once a year, and 6 = never. The responses for each item were reverse coded and rescaled (from 1–6 to 0–5) with 0 indicating never. These responses were then summed to create a continuous scale with higher values representing greater discrimination with a range of [0–34] (Cronbach’s alpha = 0.916). The everyday discrimination scale has been widely used in Black and Latinx study populations and has demonstrated high levels of validity and reliability [47].

Heightened vigilance was measured using four questions. The four-item heightened vigilance scale is a modified version of the six-item vigilance scale developed for the 1995 Detroit Area Study [48]. However, it has been used in subsequent studies assessing the influence of vigilance on health [49, 50]. Although, there was no specific mention of race in the questions, they were asked immediately following the questions about unfair treatment. Respondents were asked in their day-to-day lives, how often did they do the following things: (1) try to prepare for possible insults from other people before leaving home, (2) feel that you always have to be very careful about your appearance (to get good service or avoid being harassed), (3) carefully watch what you say and how you say it, and (4) try to avoid certain social situations and places. Responses were on a Likert-like scale of 1 = almost everyday, 2 = at least once a week, 3 = a few times a month, 4 = a few times a year, 5 = less than once a year, and 6 = never. The responses for each item were reverse coded and rescaled (from 1–6 to 0–5) with 0 indicating never. These responses were then summed to create a continuous scale with higher values representing higher levels of vigilance with a range of [0–20] (Cronbach’s alpha = 0.842).

Housing unaffordability was measured from responses to the following question: During the last 12 months, have you moved from a place because you could not afford the rent payments? During the last 12 months, have you moved from a place because you thought that if you did not move you would be evicted? Responses were dichotomous and rescaled (from 1–2 to 0–1) with 1 indicating yes. Financial strain was measured using three questions. Respondents were asked during the last 12 months: (1) How often do you personally worry because you cannot keep up with your rent payments? (2) How often do you put off buying something you need—such as food, clothing, medical care, or housing—because you do not have money? (3) How much difficulty did you have paying bills? Responses were on a Likert-like scale for each question. For question 1, the responses were 1 = worry very often, 2 = worry somewhat often, 3 = worry from time to time, and 4 = almost never worry. These responses were reverse coded and rescaled (from 1–4 to 0–3). For question 2, the responses were 1 = never, 2 = rarely, 3 = occasionally, 4 = frequently, and 5 = all of the time. Responses were rescaled from 1–5 to 0–3 by combining frequently and all of the time. For question 3, the responses were 1 = no difficulty at all, 2 = a little difficulty, 3 = some difficulty, 4 = quite a bit of difficulty, and 5 = a great deal of difficulty. Responses were rescaled from 1–5 to 0–3 by combining quite a bit of difficulty and a great deal of difficulty. These responses were then summed to create a continuous scale with higher values representing higher levels of financial strain with a range of [0–9] (Cronbach’s alpha = 0.899). We operationalized the financial strain measure into four categories (none, low, medium, high) to reflect the importance of financial strain as follows: those who reported “almost never worry,” “never,” and “no difficulty at all” on all three items were categorized as “none”; those who reported at least “worry very often,” “all of the time,” or “a great deal of difficulty” on at least one item were categorized as “high”; those who reported “worry from time to time,” “occasionally,” or “some difficulty” on at least two items were categorized as “medium”; then all others were categorized as “low.”

### Covariates

Covariates were selected based on previous literature identifying them as conceptual or empirical correlates of urban redevelopment, social environmental stress, and/or health among Black individuals [51, 52]. New development was measured from the response to “How much to do you agree or disagree with the statement: new developments within my neighborhood fit with what’s already here.” Responses were on a Likert scale of 1 = strongly agree to 5 = strongly disagree. The responses were rescaled (from 1–5 to 1–3), where 1 indicates agree and 3 indicates disagree, and then dichotomized (1 = agree vs. 0 = neither agree or disagree and disagree). Neighborhood improved was assessed from the response to the following question: Over the past year, do you think your neighborhood has … 1 = improved, 2 = stayed the same, or 3 = declined. The responses for the latter question were dichotomized (1 = improved vs. 0 = stayed the same and declined). Sociodemographic covariates including age, which was categorized into 5-year groups (from 18–22 to 83 and over), gender (1 = men vs. 0 = women and gender variant/nonconforming), and educational attainment were assessed via self-report. Educational attainment, which was rescaled as a dichotomous variable (1 = greater than a high school diploma vs. 0 = high school diploma or less), was used as a proxy for socioeconomic status.

### Analytic approach

For descriptive statistics, we estimated means with standard deviations of continuous variables and percentages of categorical variables in the total sample and by participants exposed and unexposed to greenspace redevelopment. All analyses accounted for complex two-stage research design with clustering at the block group and individual levels using Stata “svy” command. We used *t* and *χ*^*2*^ tests to test for differences by exposed and unexposed participants. We performed linear regression models to evaluate associations between subjective sleep quality and social environmental stressors (individually and combined), controlled for demographic features of study participants. The fully adjusted regression models evaluated the relationship between subjective sleep quality and social environmental stressors separately and with the respective interaction variable (e.g., financial strain × exposed), using adjusted Wald statistics. The combined model included all the social environmental stressors in the same model. We ran Scheffé’s test for post hoc pairwise comparisons of our interactions. All analyses were conducted using Stata software version 14.2 (StataCorp LLC, College Station, Texas). An alpha level of 0.05 was used for all analyses.

## Results

Descriptive statistics for subjective sleep quality, social environmental stressors, and covariates are stratified by exposed and unexposed participants (Table 1). In general, participants report poor subjective sleep quality (mean = 8.57, SD = 3.94) and there are no significant differences between exposed and unexposed participants (*p* = 0.27). Among the social environmental stressors, there are no significant differences between exposed and unexposed participants. Fifty-four percent of participants were 18–47 years of age. Forty-one percent of our samples were men. Sixty-seven percent of our sample attained more than a high school diploma. Forty-seven percent of our sample stated in the past year, their neighborhood has improved and agreed that the new developments fit within the existing neighborhood.

**Table 1:** Characteristics of total study participants and by participants exposed and unexposed to greenspace redevelopment: African American Sleep & Health Study (June–October 2019).

Sample characteristics	Total (*n* = 66)		Exposed (*n* = 24)		Unexposed (*n* = 42)		*p*
	%	Mean (SD)	%	Mean (SD)	%	Mean (SD)	
Subjective sleep quality^a^		8.57 (3.94)		9.29 (4.24)		8.15 (3.69)	0.27
Social environmental stressors							
Everyday discrimination scale		11.71 (10.10)		11.54 (10.27)		11.80 (9.99)	0.92
Heightened vigilance scale		9.00 (6.65)		8.04 (6.42)		9.58 (6.71)	0.40
Housing unaffordability							
Moved due to concerns of afford rent	10.26		20.00		4.17		0.20
Moved due to concerns of eviction	7.69		13.33		4.17		0.36
Financial strain							0.30
None	22.22		33.33		14.81		
Low	31.11		22.22		37.04		
Medium	11.11		16.67		7.41		
High	35.56		27.78		40.74		
Covariates							
Age (years)							0.16
18–22	3.08		4.17		2.44		
23–27	7.69		4.17		9.76		
28–32	15.38		16.67		14.63		
33–37	10.77		20.83		4.88		
38–42	4.62		4.17		4.88		
43–47	12.31		4.17		17.07		
48–52	9.23		4.17		12.20		
53–57	7.69		16.67		2.44		
58–62	7.69		12.50		4.88		
63–67	3.08		0		4.88		
68–72	9.23		4.17		12.20		
73–77	6.15		4.17		7.32		
83 and over	3.08		4.17		2.44		
Gender (men)	40.63		45.83		37.50		0.52
Education (higher than HS diploma)	67.19		66.67		67.50		0.95
New development fits neighborhood	46.88		43.48		48.78		0.65
Neighborhood Improved	46.88		54.17		42.50		0.40

Note all estimates account for cluster sampling design. Percentages may not sum to 100 due to missing values or rounding. *p* values result from *t*-tests (two tailed) and chi-square tests for differences between participants exposed and unexposed to greenspace redevelopment.

*HS* high school diploma, *SD* standard deviation.

^a^Subjective sleep quality = scores on the Pittsburgh Sleep Quality Index.

### Relationship between subjective sleep quality and individual social environmental stressors

The fully adjusted regression models for everyday discrimination (Model 1), heightened vigilance (Model 2), housing unaffordability (Model 3), and financial strain (Model 4) with and without interaction of being exposed to greenspace redevelopment are reported in Table 2. The independent relationships between everyday discrimination, heightened vigilance, housing unaffordability, and subjective sleep quality are not modified by being exposed to greenspace redevelopment, when controlling for other factors. One-unit increase in experiencing everyday discrimination is associated with 0.17 (CI: 0.02, 0.33, *p* < 0.05) increase in poorer subjective sleep quality. This association increased to 0.19 (CI: 0.01, 0.38, *p* < 0.05) when the interaction between everyday discrimination and exposure to greenspace redevelopment was included. Similarly, one-unit increase in experiencing heightened vigilance is associated with 0.26 (CI: 0.01, 0.51, *p* < 0.05) increase in poorer subjective sleep quality. However, this association is no longer significant when the interaction was included in the model. The association between moving due to concerns of eviction is associated with a 6.07 point (CI: 0.36, 11.77, *p* < 0.05) increase in poorer subjective sleep quality, when controlling for other factors. This association increased to 9.43 (CI: 4.53, 14.33, *p* < 0.01) when the interaction between moving due to concerns of eviction and exposure to greenspace redevelopment was included. The association between financial strain and subjective sleep quality is different for participants who are exposed and unexposed to greenspace redevelopment, while controlling for other factors, with margin values of 7.52 for participants unexposed (CI: 5.80, 9.23, *p* < 0.001) and 8.37 for exposed (CI: 4.92, 11.82, *p* < 0.01) to greenspace redevelopment (Supplementary Table 4). For exposed participants experiencing medium financial strain, their sleep quality is 12.79 points (CI: 1.36, 24.21, *p* < 0.05) poorer than exposed participants experiencing no financial strain. The Scheffé test for post hoc pairwise comparisons identified there are no statistically significant differences between the categories of financial strain and participants who are exposed and unexposed to greenspace redevelopment (Table 3).

**Table 2:** Linear regression model results for social environmental stressors, with and without exposure to greenspace redevelopment interaction, on subjective sleep quality.

	Model 1		Model 2		Model 3			Model 4		Model 5
	β (95% CI)	β (95% CI)^a^	β (95% CI)	β (95% CI)^a^	β (95% CI)	β (95% CI)^a^	β (95% CI)^a^	β (95% CI)	β (95% CI)^a^	β (95% CI)^a^
Explanatory variables										
Everyday discrimination	0.17 (0.02, 0.33)*	0.19 (0.01, 0.38)*								0.22 (0.10, 0.33)**
Everyday discrimination × exposed		−0.05 (−0.29 0.19)								
Heightened vigilance			0.26 (0.01, 0.51)*	0.27 (−0.04, 0.57)						0.05 (−0.08, 0.17)
Heightened vigilance × exposed				−0.01 (−0.44, 0.42)						
Housing unaffordability										
Moved due to concerns of afford rent					−0.07 (−6.75, 6.61)	5.16 (−1.99. 12.31)	2.50 (−2.36, 7.36)			−7.75 (−10.57, −4.93)**
Moved due to concerns of eviction					6.07 (0.36, 11.77)*	6.07 (0.36, 11.77)*	9.43 (4.53, 14.33)**			2.82 (1.20, 4.45)*
Moved due to concerns of afford rent × exposed						−5.23 (−16.41, 5.95)				
Moved due to concerns of eviction × exposed							−7.83 (−16.63, 0.97)			
Financial strain										
Low								1.90 (−4.57, 8.37)	−4.47 (−15.32, 6.38)	−14.35 (−17.04, −11.67)***
Medium								1.83 (−4.60, 8.27)	−6.79 (−18.09, 4.50)	−16.35 (−17.04, −11.67)***
High								2.63 (−2.74, 8.00)	−4.98 (−16.37, 6.38)	−17.16 (−20.15, −14.16)***
Financial strain × exposed										
Low									9.37 (−2.03, 20.76)	20.25 (16.71, 23.79)***
Medium									12.79 (1.36, 24.21)*	20.69 (17.89, 23.49)***
High									11.79 (−0.86, 24.44)	28.29 (21.82, 37.75)***
Covariates										
Exposed	1.32 (−1.44, 4.08)	1.91 (−1.57, 5.40)	0.95 (−1.81, 3.70)	1.03 (−2.72, 4.79)	−0.92 (−4.90, 3.06)	−0.92 (−4.90, 3.06)	−0.95 (−4.78, 2.87)	0.73 (−4.74, 6.21)	−7.64 (−16.89, 1.62)	−12.60 (−13.99, −11.21)***
Age										
23–27	−1.59 (−7.88, 4.71)	−1.51 (−7.89, 4.87)	−1.37(−8.23, 5.49)	−1.42 (−8.66, 5.82)	−3.27 (−11.15, 4.61)	−3.27 (−11.15, 4.61)	−2.72 (−10.38, 4.95)	−3.55 (−12.56, 5.46)	−2.46 (−10.57, 5.65)	1.29 (−2.07, 4.66)
28–32	0.76 (−3.73, 5.25)	0.89 (−3.70, 5.48)	1.46 (−3.33, 6.26)	1.42 (−3.76, 6.60)	1.06 (−5.04, 7.17)	1.06 (−5.04, 7.17)	1.14 (−4.78, 7.06)	1.36 (−5.73, 8.44)	2.24 (−4.28, 8.76)	3.07 (1.33, 4.82)*
33–37	0.74 (−5.60, 7.08)	1.14 (−5.56, 7.83)	1.09 (−5.88, 8.05)	1.08 (−5.93, 8.09)	−2.98 (−10.67, 4.70)	−2.98 (−10.67, 4.70)	−1.79 (−8.85, 5.26)	0.92 (−9.57, 11.41)	−1.36 (−12.37, 9.65)	−4.40 (−7.72, −1.08)*
38–42	−1.98 (−9.48, 5.51)	−1.69 (−9.42, 6.04)	−2.31 (−9.83, 5.21)	−2.36 (−10.44, 5.71)	−2.21 (−10.47, 6.06)	−2.21 (−10.47, 6.06)	−1.62 (−9.70, 6.46)	−1.80 (−12.13, 8.52)	−2.04 (−10.12, 6.04)	−1.54 (−4.93, 1.86)
43–47	2.15 (−3.44, 7.74)	2.34 (−3.38, 8.06)	0.87 (−5.26, 7.00)	0.81 (−6.20, 7.81)	−1.911 (−7.49, 3.67)	−1.911 (−7.49, 3.67)	−2.30 (−7.70, 3.10)	0.25 (−9.89, 10.39)	0.58 (−8.06, 9.22)	2.43 (−0.12, 4.99)
48–52	1.81 (−4.64, 8.25)	2.01 (−4.66, 8.68)	1.89 (−5.00, 8.78)	1.84 (−5.21, 8.90)	0.87 (−5.46, 7.19)	0.87 (−5.46, 7.19)	1.22 (−5.05, 7.49)	0.60 (−8.06, 9.26)	−1.29 (−7.81, 5.22)	0.64 (−1.33, 2.63)
53–57	−1.19 (−7.81, 5.44)	−0.91 (−7.82, 6.00)	0.24 (−6.52, 7.00)	0.21 (−6.56, 6.98)	−0.86 (−6.93, 5.21)	−0.86 (−6.93, 5.21)	−0.78 (−6.98, 5.43)	0.53 (−9.49, 10.55)	−0.46 (−8.38, 7.46)	−4.57 (−7.18, −1.95)*
58–62	−2.17 (−6.66, 2.33)	−1.91 (6.77, 2.95)	−1.25 (−5.50, 3.00)	−1.27 (−5.66, 3.12)	−0.83 (−6.80, 5.14)	−0.83 (−6.80, 5.14)	−0.81 (−6.80, 5.14)	−0.52 (−7.26, 6.23)	−0.58 (−9.04, 7.88)	−10.02 (−16.45, −3.59)*
63–67	0.38 (−6.20, 6.95)	0.64 (−6.11, 7.38)	−4.50 (−11.71, 2.71)	−4.58 (−12.80, 3.64)	NE	NE	NE	−2.40 (−11.83, 7.03)	−1.23 (−9.26, 6.80)	NE
68–72	2.15 (−5.36, 9.65)	2.46 (−5.15, 10.06)	2.30 (−4.82, 9.43)	2.29 (−4.98, 9.55)	5.23 (−5.95, 16.41)	5.23 (−5.95, 16.41)	NE	1.16 (−11.48, 13.80)	−1.20 (−12.09, 9.69)	13.37 (10.45, 16.28)***
73–77	0.60 (−6.46, 7.65)	0.88 (−6.27, 8.02)	0.84 (−6.52, 8.20)	0.81 (−6.68, 8.31)	3.08 (−2.97, 9.13)	3.08 (−2.97, 9.13)	3.08 (−2.97, 9.13)	−2.10 (−12.99, 8.79)	−0.75 (−9.78, 8.29)	NE
83 and over	−0.07 (−6.68, 6.55)	0.28 (−6.84, 7.41)	0.87 (−5.43, 7.16)	0.86 (−5.52, 7.23)	NE	NE	NE	1.48 (−12.63, 15.59)	−4.13 (−19.50, 11.25)	NE
Gender (men)	−1.72 (−4.65, 1.21)	−1.54 (−4.74, 1.65)	−1.79 (−4.69, 1.12)	−1.77 (−4.78, 1.23)	0.08 (−3.54, 3.71)	0.08 (−3.54, 3.71)	0.08 (−3.54, 3.71)	−1.04 (−5.53, 3.45)	−3.25 (−6.99, 0.50)	−4.95 (−6.07, −3.83)***
Education (higher than HS diploma)	−1.43 (−5.10, 2.25)	−1.48 (−5.16, 2.20)	−0.13 (−4.33, 4.08)	−0.13 (−4.33, 4.08)	0.22 (−5.03, 5.46)	0.22 (−5.03, 5.46)	0.22 (−5.03, 5.46)	0.05 (−7.10, 7.20)	−0.76 (−6.79, 5.26)	−3.02 (−5.06, −0.97)*
New development fits neighborhood	−3.18 (−6.52, 0.16)	−3.12 (−6.40, 0.17)	−3.85* (−7.24, −0.47)	−3.84* (−7.28, −0.40)	−2.79 (−7.17, 1.59)	−2.79 (−7.17, 1.59)	−2.79 (−7.17, 1.59)	−3.34 (−8.39, 1.71)	−5.73* (−9.80, −1.67)	−8.15 (−9.25, −7.05)***
Neighborhood improved	1.71 (−1.36, 4.78)	1.74 (−1.29, 4.76)	2.41 (−0.78, 5.60)	2.41 (−0.86, 5.67)	2.02 (−5.03, 5.46)	2.02 (−1.29, 5.33)	2.02 (−1.29, 5.33)	0.94 (−3.03, 4.91)	1.60 (−2.06, 5.26)	0.59 (−0.59, 1.78)
Constant	8.02 (2.33, 13.71)*	7.49 (1.21, 13.77)*	6.77 (0.23, 13.30)*	6.76 (0.20, 13.32)*	9.62, (3.10, 16.15)*	9.62 (3.10, 16.15)*	9.62, (3.10, 16.15)*	7.82 (−3.99, 19.62)	16.62 (1.72, 31.52)*	24.84 (21.05, 28.62)***
Observations	61	61	60	60	37	37	37	43	43	30
R^2^	0.43	0.44	0.42	0.42	0.52	0.52	0.55	0.40	0.55	0.99
Adjusted Wald test	**	*	*		*		*	**	**	***

Note all estimates account for cluster sampling design. Differences in observations are due to missing data. For Model 3, the sample size is smaller due to this question only being asked to renters and not homeowners.

*NE* not estimable, *CI* confidence intervals.

β = regression coefficients; × represents interaction between social environmental stressor and being exposed to greenspace redevelopment.

*p *<* 0.05; **p *<* 0.01; ***p *<* 0.001.

^a^Models with interaction variable (i.e., exposed to greenspace redevelopment).

**Table 3:** Adjusted mean differences between pairwise comparisons of financial strain model (Model 4) and combined social environmental stressors model (Model 5).

Explanatory variables	Model 4	Model 5
	M_Diff_ (95% CI)	M_Diff_ (95% CI)
Financial strain		
Medium vs. none	−0.40 (−11.58, 10.77)	−6.11 (−9.93, −2.29)*
Low vs. none	0.21 (−9.80, 10.23)	−4.23 (−6.88, −1.57)*
Medium vs. low	−0.61 (−6.05, 4.82)	−1.89 (−4.57, 0. 80)
High vs. none	0.92 (−10.38, 12.21)	−3.01 (−6.88, 0.86)
High vs. low	0.70 (–6.42, 7.83)	1.21 (−2.44, 4.86)
High vs. medium	1.32 (−6.29, 8.92)	3.10 (−2.52, 8.72)
Exposed	0.85 (−3.12, 4.82)	4.71 (2.94, 6.47)**
Financial strain × exposed		
(High × unexposed) vs. (None × unexposed)	−4.98 (−33.12, 23.16)	−17.16 (−24.58, −9.74)**
(Medium × unexposed) vs. (None × unexposed)	−6.79 (−34.79, 21.20)	−16.46 (−23.74, −9.18)**
(Low × unexposed) vs. (None × unexposed)	−4.47 (−31.35, 22.41)	−14.35 (−21.01, −7.70)**
(None × exposed) vs. (None × unexposed)	−7.64 (−30.57, 15.30)	−12.60 (−16.04, −9.16)***
(High × unexposed) vs. (Low × exposed)	−2.24 (−18.66, 14.18)	−10.46 (−16.12, −4.79)**
(Medium × unexposed) vs. (Low × exposed)	−4.05 (−18.18, 10.07)	−9.76 (−15.80, −3.71)*
(High × unexposed) vs. (Medium × exposed)	−3.34 (−18.09, 11.41)	−8.79 (−15.70, −1.88)*
(Medium × exposed) vs. (None × unexposed)	−1.64 (−28.25, 24.96)	−8.37 (−15.28, −1.45)*
(Low × exposed) vs. (None × unexposed)	−2.74 (−27.65, 22.17)	−6.70 (−11.54, −1.86)*
(High × unexposed) vs. (None × exposed)	2.66 (−10.60, 15.91)	−4.56 (−10.20, 1.08)
(Medium × unexposed) vs. (None × exposed)	0.84 (−12.04, 13.73)	−3.86 (−9.33, 1.61)
(High × unexposed) vs. (Low × unexposed)	−0.51 (−13.31, 12.28)	−2.81 (−7.14, 1.53)
(Medium × unexposed) vs. (Low × unexposed)	−2.32 (−15.70, 11.06)	−2.10 (−6.28, 2.07)
(Medium × exposed) vs. (Low × exposed)	1.09 (−11.33, 13.52)	−1.67 (−9.18, 5.84)
(Low × unexposed) vs. (None × exposed)	3.17 (−9.16, 15.50)	−1.75 (−6.76, 3.26)
(High × unexposed) vs. (Medium × unexposed)	1.81 (−9.23, 12.86)	−0.70 (−4.66, 3.26)
(High × exposed) vs. (None × unexposed)	−0.82 (−30.35, 28.71)	−1.47 (−11.74, 8.81)
(Medium × exposed) vs. (None × exposed)	5.99 (−7.25, 19.24)	4.23 (−1.80, 10.27)
(Medium × exposed) vs. (Low × unexposed)	2.83 (−8.08, 13.73)	5.99 (1.25, 10.72)*
(Low × exposed) vs. (None × exposed)	4.90 (−6.25, 16.05)	5.90 (0.84, 10.96)*
(Low × exposed) vs. (Low × unexposed)	1.73 (−12.30, 15.76)	7.65 (1.05, 14.26)*
(Medium × exposed) vs. (Medium × unexposed)	5.15 (−8.66, 18.96)	8.09 (3.11, 13.07)*
(High × exposed) vs. (Low × exposed)	1.92 (−12.63, 16.46)	5.23 (−4.15, 14.61)
(High × exposed) vs. (Medium × exposed)	0.82 (−20.03, 21.67)	6.90 (−8.18, 21.97)
(High × exposed) vs. (None × exposed)	6.81 (−9.62, 23.24)	11.13 (−0.71, 22.97)
(High × exposed) vs. (Low × unexposed)	3.65 (−17.52, 24.81)	12.88 (−1.63, 27.40)
(High × exposed) vs. (Medium × unexposed)	5.97 (−13.40, 25.34)	14.99 (0.71, 29.26)*
(High × exposed) vs. (High × unexposed)	4.16 (−16.87, 25.19)	15.69 (2.02, 29.36)*

Note mean differences were compared using Scheffe’s test. Exposed means participants exposed to greenspace redevelopment.

*CI* confidence intervals.

*p < 0.05; **p < 0.01; ***p < 0.001.

### Relationship between subjective sleep quality and combined social environmental stressors

The fully adjusted regression model for combined social environmental stressors (Model 5) is reported in Table 2. The subjective sleep quality score of participants who moved because they could not afford the rent payments is better by 7.75 points (CI: −10.57, −4.93, *p* < 0.01) compared to participants who did not move. The subjective sleep quality of participants who moved because of concerns of being evicted is poorer by 2.82 points (CI: 1.20, 4.45, *p* < 0.05) compared to participants who did not move. The associations between everyday discrimination, financial strain, and subjective sleep quality persisted in the combined model. One-unit increase in experiencing everyday discrimination is associated with a 0.22 increase in poorer subjective sleep quality (CI: 0.10, 0.33, *p* < 0.01). The association between financial strain and subjective sleep quality is different for participants exposed and unexposed to greenspace redevelopment, while controlling for other factors, with margin values of 10.70 points (CI: 9.76, 11.64, *p* < 0.001) for exposed and 5.99 points (CI: 3.73, 8.26, *p* < 0.01) for unexposed participants.

The Scheffé test for post hoc pairwise comparisons identified there are statistically significant differences between the categories of financial strain and participants who are exposed and unexposed to greenspace redevelopment (Table 3). For exposed participants experiencing high financial strain compared to unexposed participants experiencing high financial strain (i.e., high × exposed vs. high × unexposed), their subjective sleep quality score is poorer by 15.69 points (CI: 2.02, 29.36, *p* < 0.05). Similarly, for exposed participants experiencing high financial strain compared to unexposed participants experiencing medium financial strain (i.e., high × exposed vs. medium × unexposed), their sleep quality score is poorer 14.99 points (CI: 0.71, 29.26, *p* < 0.05). The subjective sleep quality score of exposed participants experiencing medium financial strain is poorer compared to unexposed participants experiencing medium financial strain (i.e., medium × exposed vs. medium × unexposed) by 8.09 points (CI: 3.11, 13.07, *p* < 0.05). Likewise, the subjective sleep quality score of exposed participants experiencing medium financial strain is poorer compared to unexposed participants experiencing low financial strain (i.e., medium × exposed vs. low × unexposed) by 5.99 points (CI: 1.25, 10.72, *p* < 0.05). The subjective sleep quality score of exposed participants experiencing low financial strain is poorer by 7.65 points (CI: 1.05, 14.26, *p* < 0.05) compared to unexposed participants experiencing low financial strain (i.e., low × exposed vs. low × unexposed).

Alternatively, the subjective sleep quality score of exposed participants experiencing medium financial strain is better by 8.37 points (−15.28, −1.45, *p* < 0.05) compared to unexposed participants not experiencing financial strain (i.e., medium × exposed vs. none × unexposed). For exposed participants experiencing low financial strain compared to unexposed participants not experiencing financial strain (i.e., low × exposed vs. none × unexposed), their subjective sleep quality is better by 6.70 points (−11.54, −1.86, *p* < 0.05). Similarly, the subjective sleep quality score of exposed participants not experiencing financial strain is better by 12.60 points (−16.04, −9.16, *p* < 0.001) compared to unexposed participants not experiencing financial strain (i.e., none × exposed vs. none × unexposed). For unexposed participants experiencing medium financial strain, their subjective sleep quality score is better compared to exposed participants experiencing low financial strain (i.e., medium × unexposed vs. low × exposed) by 9.76 points (CI: −15.80, −3.71, *p* < 0.05). Likewise, the subjective sleep quality score of unexposed participants experiencing high financial strain is better compared to exposed participants experiencing low financial strain (i.e., high × unexposed vs. low × exposed) by 10.46 points (CI: −16.12, −4.79, *p* < 0.01).

## Discussion

To our knowledge, this is the first study to conduct a cross-sectional, quasi-experimental study examining the relationship between social environmental stressors associated with residential displacement and sleep quality among Black adults. We found that everyday discrimination, heightened vigilance, and housing unaffordability—specifically participants who moved in the past 12 months due to concerns of being evicted—are independently associated with poorer subjective sleep quality and there are no differences between exposed and unexposed participants. In our combined model, the association between everyday discrimination and poorer sleep quality persisted and was slightly stronger, which suggests that experiences of discrimination are an important contributor to sleep quality. We also found that participants who moved in the past 12 months due to concerns of being evicted were associated with poorer subjective sleep quality, whereas participants who moved in the past 12 months because they could not afford the rent were associated with better sleep quality compared to participants who did not move. Finally, the association between financial strain and subjective sleep quality was different for participants exposed and unexposed to greenspace redevelopment, with exposed participants generally having poorer subjective sleep quality compared to their unexposed counterpart. However, there were financial strain categories where exposed participants had better sleep quality than unexposed participants.

Our results are consistent with research that has investigated the relationship between discrimination [29, 30], vigilance [33], housing insecurity [28], and sleep. Research has shown that stress related to discrimination and preparation for future experiences of discrimination (i.e., vigilance) is common for Black adults as they navigate the social spaces that are a part of their daily activities [33, 53]. Our findings also suggest that moving due to concerns of not being able to pay rent is distinct from moving due to concerns of being evicted. It may be the impetus for the move and the participant’s experience in their next residence that could be differentially impacting their self-reported sleep quality. For example, moving due to concerns of not being able to pay rent could be categorized as a “responsive move” [54, 55] because the motivation for the move is related to housing and/or neighborhood factors (e.g., increases in housing costs, deterioration in housing quality, escalating neighborhood violence). Liu et al. [28] found that individuals who reported worrying about not having enough money to pay rent (i.e., housing insecurity) were more likely to self-report frequent insufficient sleep than those who did not report housing insecurity. We postulate that participants who moved due to concerns of being able to pay rent had better sleep quality because they had greater residential stability and reduced their stress by moving into a more stable and affordable housing [56]. On the other hand, moving due to concerns of being evicted could be categorized as a “forced move” [54, 55], where renters have no other choice than to relocate (or at least believe they do not have a choice) because of the initiation of formal eviction (i.e., court ordered removal from their homes) and/or informal eviction processes (i.e., renters are forced from their homes before a formal eviction process is initiated, as well as, departures that occur after the eviction filing, but before a formal judgment occurs). The threat of eviction can create barriers to future housing security and mobility. For instance, eviction filings can effectively prevent renters from future eligibility for subsidized housing [54] and landlords could reject applicants with any negative housing history (e.g., eviction filing—even in cases where the court process did not render an eviction judgment) [57, 58]. After experiencing a forced move, Desmond and Shollenberger [55] found there was an increased chance by as much as 70% that the renter will experience housing problems in their next residence. Studies have shown an increased likelihood of developing depression symptoms, experiencing an anxiety attack, and psychological distress [59, 60] after experiencing an eviction threat. There is an association between depression, anxiety, and sleep disturbances [61].

Our findings complement and extend a previous study examining the relationship between subjective sleep quality and financial strain [27] in three ways—first we used a combined measure of financial strain to capture the economic vulnerability of study participants. Second, we investigated the relationship between financial strain and sleep within the context of greenspace redevelopment. Third, we found that the relationship between financial strain and sleep is nuanced within the context of greenspace redevelopment. For example, the subjective sleep quality of exposed participants not experiencing financial strain is better compared to unexposed participants not experiencing financial strain. This finding corroborates with research that has shown that residing in a “greener” neighborhood is associated with healthier sleep duration [4, 62]. On the other hand, exposed participants experiencing financial strain (i.e., low, medium, and high) generally had poorer subjective sleep quality compared to their unexposed counterpart. This could be attributed to neighborhood changes related to greenspace redevelopment (e.g., increases in housing costs), which could increase the pressure of displacement for exposed participants. However, for exposed participants experiencing medium, low, and no financial strain, their subjective sleep quality is better compared to unexposed participants not experiencing financial strain. We posit that although these exposed participants are experiencing at most occasional financial strain, they could also be experiencing the sleep benefits associated with the exposure to greenspace and/or there are other neighborhood social factors that are encouraging them to stay (e.g., strong social ties to the historical significance of their Black neighborhood and their remaining Black neighbors) [63]. It is hypothesized that neighborhood social cohesion could influence sleep health by mitigating the effects of stress by enhancing safety, social support, and trust among neighbors [64, 65]. Future research should conduct qualitative studies to better understand the nuanced relationship between financial strain and subjective sleep quality within the context of greenspace redevelopment.

The results of our study should be interpreted considering the following limitations. First, our measures of social environmental stressors and sleep quality were self-reported. Although there is evidence in the sleep literature that objective and subjective forms of sleep quality are similarly correlated with everyday discrimination [29] and financial strain [26], the use of objective measures, such as polysomnography, or actigraphy, is preferred, especially since PSQI does not measure regular sleep schedules. Second, our measure of everyday discrimination and heightened vigilance focused on overall discrimination, rather than cause specific (e.g., racial or gender discrimination). Therefore, it is unclear whether a cause-specific measure would have altered our results. Third, the cross-sectional nature of our study limits the ability of causal inference of the effect of social environmental stressors on sleep quality. Furthermore, the temporal dynamics around the cross-sectional association between social environmental stressors and sleep quality are unclear. For example, reports of discrimination, heightened vigilance, housing unaffordability, and financial strain are experiences that occurred within the past 12 months, while sleep reflects the past month. The use of longitudinal data in future studies would strengthen our confidence in the proposed causal directions presented in our conceptual framework. Fourth, we did not explicitly measure whether neighborhood improvements and social environmental stressors are related to the Atlanta BeltLine. Therefore, it is possible other variables not considered in our model may explain our empirical results. Fifth, although the AASH study is community based and consists of Black adults from four census block groups in Southwest Atlanta, the small sample size and sample selection may limit our ability to generalize the results of this study to other racial groups and populations in other geographic locations is potentially weak. In addition, the small sample size was unsurprising given we were anticipating a decrease in Black residents within our exposed block groups following the completion of the Westside trail in 2017. Similarly, the low response rate was expected because residents were understandably apprehensive in opening their doors for the study team (i.e., strangers) due to local crime rates and the high frequency in which developers make offers on their homes, especially in areas identified as exposed to greenspace redevelopment (i.e., medium to high-risk areas of displacement). Lastly, missing data are a potential limitation that could affect the relationship between social environmental stressors and subjective sleep quality.

Despite the limitations of this study, we were able to investigate for the first time the relationship between social environmental stressors associated with residential displacement, independently and combined, and sleep quality among Black residents. Our findings suggest that everyday discrimination, heightened vigilance, housing unaffordability, and financial strain are important determinants of subjective sleep quality and their relationship is nuanced within the context of greenspace redevelopment. Therefore, municipalities need to consider the costs and benefits of greenspace redevelopment to communities who have historically and disproportionately lived in disinvested environments due to residential segregation. Without the implementation of policies to mitigate the potential economic and social stressors associated with greenspace redevelopment—such as supporting current renters through rent stabilization measures and prioritizing the rehabilitation, preservation, and inclusion of affordable housing in areas targeted for redevelopment activity—neighborhood changes can amplify existing social environmental stressors. As shown with a key health behavior, such as subjective sleep quality, these stressors can have negative health implications. Future research should replicate these analyses within a larger participant sample, conduct qualitative interviews to gain greater insight on the nuances associated with financial strain and sleep quality within the context of greenspace redevelopment, and explore prospective longitudinal studies that can examine the temporal order, extent, and severity of greenspace redevelopment efforts on social environmental stressors and sleep quality. A greater understanding of the temporal ordering, extent, and severity of these associations will have implications for city planning and public health strategies that are focused on creating healthier and more equitable communities.

## Supplementary information

Supplemetary Figure 1

Supplementary Table 1

Supplementary Table 2

Supplementary Table 3

Supplementary Table 4
