# Supplementary figures and images for: Greenspace redevelopment, pressure of displacement, and sleep quality among Black adults in Southwest Atlanta
Source: J Expo Sci Environ Epidemiol. 2021 Mar 13;31(3):412–26. doi: 10.1038/s41370-021-00313-9 (PMC8134046; doi:10.1038/s41370-021-00313-9)

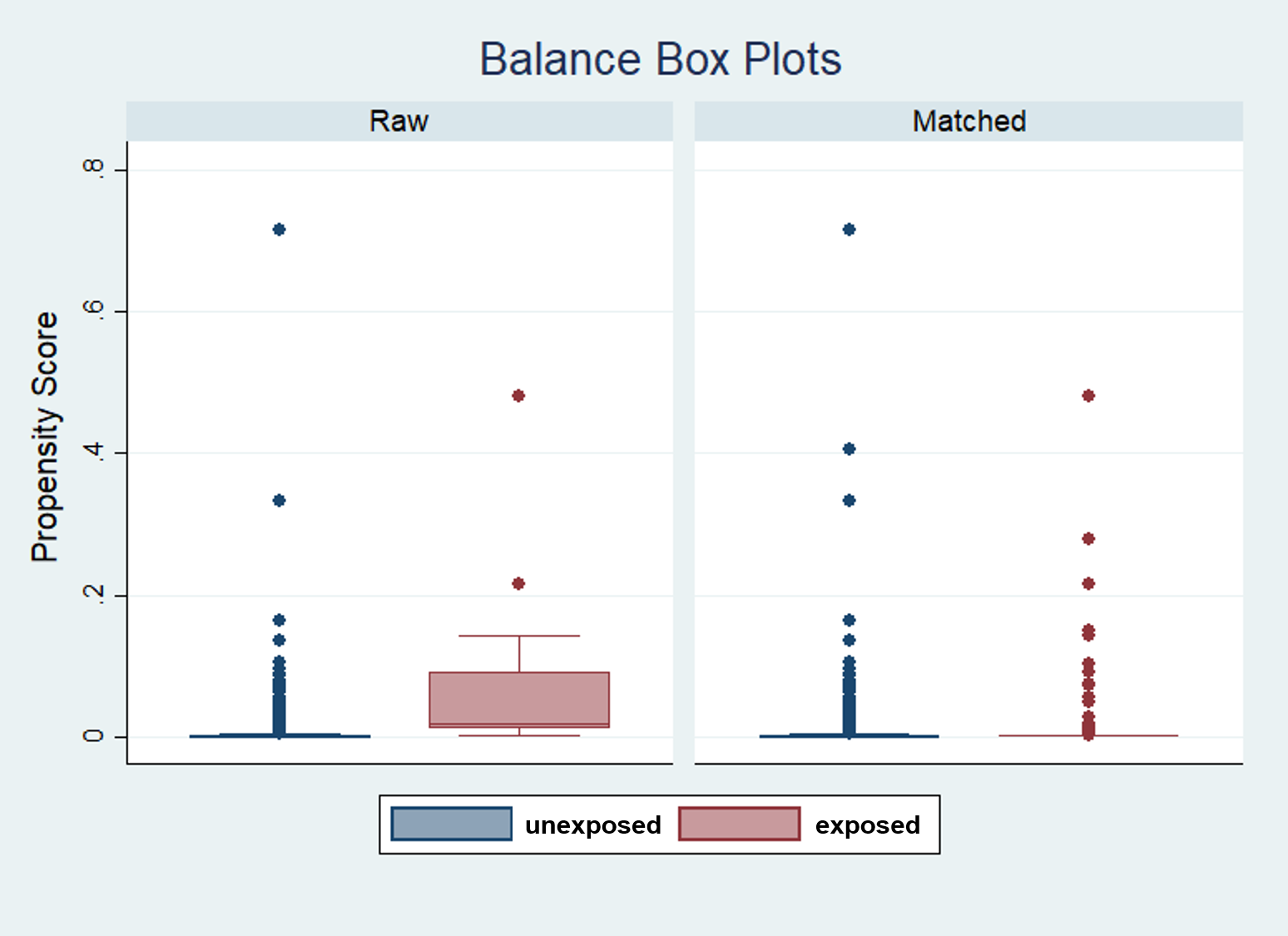

Supplement: Supplementary file 1 — Supplemetary Figure 1 [file 41370_2021_313_MOESM1_ESM.tif]
