# Supplementary material for: Greenspace redevelopment, pressure of displacement, and sleep quality among Black adults in Southwest Atlanta
Source: J Expo Sci Environ Epidemiol. 2021 Mar 13;31(3):412–26. doi: 10.1038/s41370-021-00313-9 (PMC8134046; doi:10.1038/s41370-021-00313-9)
Supplement: Supplementary file 2 — Supplementary Table 1 [file 41370_2021_313_MOESM2_ESM.docx]

Supplementary Table 1. Displacement Risk Indicators

| Vulnerability Index | Description |
| --- | --- |
| NH-African American/Black | Percentage of population who are Non-Hispanic African American/Black |
| Hispanic | Percentage of population who are Hispanic |
| NH-American Indian and Alaskan Native | Percentage of population who are Non-Hispanic American Indian and Alaskan Native |
| NH-Asian | Percentage of population who are Non-Hispanic Asian |
| NH-Native Hawaiian and Other Pacific Islander | Percentage of population who are Non-Hispanic Native Hawaiian and Other Pacific Islander |
| Elderly | Percentage of population who are 65 years or older without a disability |
| Single-parent householders | Percentage of male and female householders with related children under 18 years |
| English speaking ability | Percentage of population 5 years and older who speak English less than “very well” |
| Educational Attainment | Percentage of population 25 years or older who only have a high school diploma or the equivalent |
| Housing Tenancy | Percentage of households that are renters |
| Housing cost-burdened households | Percentage of renters paying between 30% and 49.9% of household income on gross rent in the past 12 months and homeowners, with and without a mortgage, paying between 30% and 49.9% of household income on monthly owner costs in the past 12 months |
| Housing severely-cost-burdened households | Percentage of renters paying greater than 50% of household income on gross rent in the past 12 months and homeowners, with and without a mortgage, paying greater than 50% of household income on monthly owner costs in the past 12 months |
| Household income | Percentage of population with income below poverty level |
| Housing Market Index | **Description** |
| Change in Affordable Housing Units | The number of affordable housing units lost/gained from previous year per renter-occupied units |
| Expiring Affordable Housing Subsidies | The number of affordable housing unit subsidies that are due to expire per renter-occupied units |
| Median Home Value | Median home value |
| Median Rent | Median gross rent |
| Crime rate | Number of crimes reported per total population |
| Eviction rate | Number of evictions per renter-occupied households |
| School Poverty Status | Percent of students eligible for free or reduced lunch |
| Vacancy rate | Percentage of total housing units that are vacant |
| Lower-income area next to higher-income area | Below-median income areas adjacent to above-median income areas |
