# Supplementary material for: Greenspace redevelopment, pressure of displacement, and sleep quality among Black adults in Southwest Atlanta
Source: J Expo Sci Environ Epidemiol. 2021 Mar 13;31(3):412–26. doi: 10.1038/s41370-021-00313-9 (PMC8134046; doi:10.1038/s41370-021-00313-9)
Supplement: Supplementary file 3 — Supplementary Table 2 [file 41370_2021_313_MOESM3_ESM.docx]

| Racial, Economic, and Housing Indicators | Exposed Block Group #1 | Unexposed Block Group #1 | Exposed Block Group #2 | Unexposed Block Group #2 |
| --- | --- | --- | --- | --- |
| Percent Below Poverty | 33.85% | 28.88% | 47.80% | 46.53% |
| Percent of population who are Non-Hispanic African American/Black | 100% | 100% | 100% | 100% |
| Percent of population who are Non-Hispanic White | 0% | 0% | 0% | 0% |
| Percent of households who are renters | 70.77% | 41.42% | 76.92% | 91.22% |
| Percent of population in management occupations | 0% | 4.33% | 0% | 0% |
| Percent of population in service occupations | 32.92% | 18.50% | 25.32% | 24.32% |
| Median Home Value | $43,900 | $86,800 | $55,800 | $52,900 |
| Median Rent | $1,170 | $1,036 | $1,124 | $785 |
| Percent of population with bachelor’s degree or higher | 9.79% | 29.97% | 2.47% | 7.40% |
| Median Household Income | $12,987 | $4,561 | $0 | $5,821 |
| Unemployment Rate | 6.96% | 11.33% | 14.62% | 15.31% |
| Percent of population who are elderly | 12.84% | 24.06% | 13.49% | 6.94% |
| Percent of population female householders | 15.06% | 27.91% | 24.77% | 23.92% |
| Propensity Scores | 0.012 | 0.012 | 0.055 | 0.054 |

Supplementary Table 2. Descriptive Characteristics of Exposed and Unexposed Block Groups Matched using Propensity Score Matching

Note data is from the 2012-2016 American Community Survey 5-year estimates
