# Supplementary material for: Greenspace redevelopment, pressure of displacement, and sleep quality among Black adults in Southwest Atlanta
Source: J Expo Sci Environ Epidemiol. 2021 Mar 13;31(3):412–26. doi: 10.1038/s41370-021-00313-9 (PMC8134046; doi:10.1038/s41370-021-00313-9)
Supplement: Supplementary file 4 — Supplementary Table 3 [file 41370_2021_313_MOESM4_ESM.docx]

| Variable | Original Sample | | | Propensity Score Matched Sample | | |
| --- | --- | --- | --- | --- | --- | --- |
|  | **Mean Exposed (n = 13)** | **Mean Comparison (n = 5507)** | **Standardized Difference** | **Mean Exposed (n = 13)** | **Mean Comparison (n = 4484)** | **Standardized Difference** |
| Percent of population who are Non-Hispanic African American/Black | 0.627 | 0.323 | 0.808 | 0.627 | 0.378 | -0.042 |
| Percent of population who are Non-Hispanic White | 0.262 | 0.543 | -0.790 | 0.262 | 0.480 | 0.028 |
| Percent of households who are renters | 0.756 | 0.382 | 1.700 | 0.756 | 0.433 | 1.362 |
| Percent of population in management occupations | 0.073 | 0.094 | -0.192 | 0.073 | 0.088 | 0.553 |
| Percent of population in service occupations | 0.191 | 0.181 | 0.032 | 0.191 | 0.188 | -0.072 |
| Median Home Value | 249384.60 | 146489.30 | 0.637 | 249384.60 | 144437.00 | 0.683 |
| Median Rent | 926.85 | 783.62 | 0.156 | 926.85 | 875.62 | -0.074 |
| Percent of population with bachelor’s degree or higher | 0.357 | 0.269 | 0.407 | 0.357 | 0.270 | 0.585 |
| Median Household Income | 10647.92 | 15394.32 | -0.392 | 10647.92 | 13748.58 | -0.004 |
| Unemployment Rate | 0.078 | 0.056 | 0.431 | 0.078 | 0.059 | -0.076 |
| Percent of population who are elderly | 0.111 | 0.138 | -0.267 | 0.111 | 0.132 | -1.045 |
| Percent of population female householders | 0.253 | 0.190 | 0.809 | 0.253 | 0.197 | -0.003 |

Supplementary Table 3. Covariate Balance across Exposed and Comparison Groups before and after Propensity Score Matching

Note data is from the 2012-2016 American Community Survey 5-year estimates
