# Supplementary material for: Greenspace redevelopment, pressure of displacement, and sleep quality among Black adults in Southwest Atlanta
Source: J Expo Sci Environ Epidemiol. 2021 Mar 13;31(3):412–26. doi: 10.1038/s41370-021-00313-9 (PMC8134046; doi:10.1038/s41370-021-00313-9)
Supplement: Supplementary file 5 — Supplementary Table 4 [file 41370_2021_313_MOESM5_ESM.docx]

Supplementary Table 4. Predictive Margins of Financial Strain Model (Model 4) and Combined Social Environmental Stressors Model (Model 5)

| Explanatory Variables | | Model 4 | Model 5 |
| --- | --- | --- | --- |
|  |  | Predictive Margin (95% CI) | Predictive Margin (95% CI) |
| Financial Strain | |  |  |
|  | None | 7.76 (2.87, 12.65) * | 11.68 (10.08, 13.29) *** |
|  | Low | 7.97 (4.56, 11.39) ** | 7.46 (5.53, 9.38) *** |
|  | Medium | 7.36 (4.02, 10.70) ** | 5.57 (2.52, 8.63) ** |
|  | High | 8.68 (5.23, 12.12) ** | 8.67 (7.50, 9.85) *** |
| Exposed | |  |  |
|  | Unexposed | 7.52 (5.80, 9.23) *** | 5.99 (3.73, 8.26) ** |
|  | Exposed | 8.37 (4.92, 11.82) ** | 10.70 (9.76, 11.64) *** |
| Financial Strain X Exposed | |  |  |
|  | None X unexposed | 11.58 (2.66, 20.50) * | 17.98 (16.48, 19.49) *** |
|  | None X exposed | 3.94 (0.61, 7.27) * | 5.39 (3.43, 7.34) ** |
|  | Low X unexposed | 7.11 (2.86, 11.36) * | 3.63 (0.63, 6.64) * |
|  | Low X exposed | 8.84 (4.22, 13.46) ** | 11.28 (9.90, 12.67) *** |
|  | Medium X unexposed | 4.78 (1.21, 8.36) * | 1.53 (-1.50, 4.56) |
|  | Medium X exposed | 9.93 (4.93, 14.94) ** | 9.62 (6.23, 13.01) ** |
|  | High X unexposed | 6.60 (2.87, 10.32) * | 0.83 (-2.06, 3.72) |
|  | High X exposed | 10.75 (3.98, 17.53) * | 16.52 (13.41, 19.62) *** |

* p < 0.05, ** p < 0.01, *** p <0.001; CI = Confidence Intervals

Exposed means participants exposed to greenspace redevelopment
